# The Impact of Elevated Lipoprotein (a) Levels on Postoperative Outcomes in Carotid Endarterectomy: A Systematic Review

**DOI:** 10.3390/jcm14072253

**Published:** 2025-03-26

**Authors:** João Carvalheiras Marques, Mariana Fragão Marques, Hugo Ribeiro, António Pereira Neves, Peter Zlatanovic, João Rocha Neves

**Affiliations:** 1 Faculty of Medicine, University of Porto, 4200-319 Porto, Portugal; marianaif.rm@gmail.com (M.F.M.); hribeiroff@gmail.com (H.R.); 2 Community Palliative Care Support Team Gaia, Health Local Unit Gaia and Espinho, 4434-502 Vila Nova de Gaia, Portugal; 3 Faculty of Medicine, University of Coimbra, 3000-370 Coimbra, Portugal; 4 Coimbra Institute for Biomedical Research, 3000-548 Coimbra, Portugal; 5 Department of Vascular Surgery, Health Local Unit of São João, 4200-319 Porto, Portugal; antonio.hpneves@gmail.com; 6 Department of Biomedicine, Unity of Anatomy, Faculty of Medicine, University of Porto, 4200-319 Porto, Portugal; joaorochaneves@hotmail.com; 7 Clinic for Vascular and Endovascular Surgery, University Clinical Centre of Serbia, 11000 Belgrade, Serbia; petar91goldy@gmail.com; 8 RISE-Health, Department of Biomedicine, Faculty of Medicine, University of Porto, Alameda Prof. Hernâni Monteiro, 4200-319 Porto, Portugal

**Keywords:** thrombosis, dyslipidemia, carotid stenosis, cardiovascular diseases, stroke, cardiovascular risk factor

## Abstract

**Background/Objectives:** Numerous studies have highlighted lipoprotein (a) (Lp(a)) as a significant, independent risk factor for the development and progression of cardiovascular diseases, including carotid artery disease, which is strongly correlated with an elevated risk of ischemic events and stroke. This systematic review aims to determine the impact of elevated Lp(a) levels on the postoperative outcomes in patients undergoing carotid endarterectomy (CEA). **Methods**: Four electronic databases—PubMed, Scopus, Web of Science, and Cochrane Library—were employed to search for studies assessing the association between elevated Lp(a) levels and the postoperative outcomes following CEA. The effect of elevated Lp(a) levels was systematically reviewed, and the outcomes reported in each study were evaluated. The quality of the studies was evaluated using the National Heart, Lung, and Blood Institute Study Quality Assessment Tool for observational cohorts and cross-sectional studies. **Results**: A total of five observational studies were included, with 1450 patients. The mean age of the participants in the studies ranged from 57 to 74 years, and the percentage of males ranged from 37.22% to 68.96%. One study showed that elevated Lp(a) levels were significantly associated with major adverse cardiovascular events (MACEs) after CEA, particularly periprocedural stroke, with another manuscript suggesting a long-term predictive value for acute coronary syndromes (ACSs) within 24 months following surgery. There was no association in the included studies with carotid plaque instability, inflammation biomarkers, or restenosis. **Conclusions**: This systematic review suggests an association of Lp(a) levels with MACEs and ACSs after CEA although no association with restenosis and carotid plaque inflammation and/or instability.

## 1. Introduction

Carotid endarterectomy is a surgical procedure performed on patients with atherosclerotic lesions to address stenosis, ensuring an adequate blood flow while minimizing the risk of plaque debris embolization. This technique involves excising the atherosclerotic plaque that partially obstructs the blood flow with the primary objective of preventing ischemic stroke due to ipsilateral carotid disease [1]. Currently, there are indications for performing CEA in both asymptomatic patients—those with stenosis between 60 and 99%, a life expectancy of over five years, and at least one additional stroke risk factor—and symptomatic patients—with stenosis between 50 and 99% with the best medical therapy (BMT) already applied [2]. CEA is the standard treatment when it comes to preventing strokes in symptomatic or asymptomatic patients and has favorable perioperative and long-term (>5-year) outcomes [3].

Lp(a) is a low-density lipoprotein (LDL)-like particle formed through a covalent bond between Apo(a) (apolipoprotein(a)) and Apo B100 (apolipoprotein B100) of LDL. Like LDL cholesterol, Lp(a) can accumulate in the subendothelial space, promoting the progression of atherosclerotic lesions. It induces a systemic pro-inflammatory state and exhibits prothrombotic and pro-oxidant properties, significantly contributing to vascular damage and thromboembolic complications [4,5,6,7]. Lp(a) contains a structural component similar to plasminogen, Apo(a), which leads to reduced fibrinolytic activity. This reduction limits efficient thrombus degradation, subsequently increasing the risk of ischemic events [4]. Furthermore, previous studies have identified Lp(a) as an independent risk factor for the development of peripheral vascular disease (PAD) and coronary artery disease (CAD) and the recurrence of cardiovascular events such as stroke and acute myocardial infarction [4,8,9].

Other than Lp(a), studies have demonstrated that other biomarkers may be involved in atherosclerotic plaques, such as oxidized low-density lipoprotein (ox-LDL), matrix metalloproteinase-9 (MMP-9), and 8-hydroxy-2′-deoxyguanosine (8-OHdG). Recent research has shown that ox-LDL plays a relevant role in the formation of atherosclerotic lesions and indirectly influences the expression and activity of MMPs, particularly MMP-9, within atherosclerotic plaques [10]. Furthermore, ox-LDL can activate monocyte chemoattractant protein-1 (MCP-1), leading to endothelial damage and neovascularization [11]. MMP-9 has frequently been associated with cardiovascular pathology and may hold clinical significance in the pathogenesis of atherosclerosis [12]. Additionally, 8-OHdG, a biomarker of oxidative DNA damage, has been linked to endothelial dysfunction and atherosclerotic lesions, with elevated levels correlating with the number of vessels affected by atherosclerosis and contributing to vascular disease progression through cumulative DNA damage [13,14].

Thus, this systematic review aims to evaluate the impact of elevated Lp(a) levels on the postoperative outcomes in patients undergoing CEA. Given Lp(a)’s role in promoting atherosclerotic lesion progression, inducing a pro-inflammatory and prothrombotic state, and impairing fibrinolysis due to its structural similarity to plasminogen, it emerges as an independent risk factor for cardiovascular events. Understanding its influence on the surgical outcomes following CEA could provide valuable insights into the patient risk stratification and guide perioperative management strategies.

## 2. Materials and Methods

This systematic review was conducted according to the Preferred Reporting Items for a Systematic Review and Meta-analysis (PRISMA) Statement [15] and the AMSTAR -2 critical appraisal tool [16]. The details of the PRISMA checklist are described in Supplemental Table S1. Ethical approval from an institutional review board was not required given this study’s design and the nature of the data analyzed. The review protocol was registered on Prospero (reference: CRD42024603037).

### 2.1. The Selection Criteria

The inclusion criteria comprised all original studies conducted in humans, excluding systematic reviews, case series, and case reports, in which the impact of elevated Lp(a) levels on the postoperative outcomes in patients undergoing CEA was assessed. The exclusion criteria included patients undergoing second surgery due to restenosis and endovascular surgery, including carotid stenting. No exclusion criteria were applied concerning the language or the date of publication.

### 2.2. The Search Strategy

A systematic search was performed in four databases—PubMed, Web of Science, Scopus, and Cochrane Library—in October 2024. The query is shown in Supplemental Table S2. Furthermore, the references of the included studies were reviewed in full to identify any additional articles of potential interest.

### 2.3. Study Selection and Data Extraction

After removing duplicates, two authors (J.C.M. and J.R.N.) independently selected the studies, with any disagreements resolved by a third author (A.P.N.). The initial screening was based on their titles and abstracts, and studies that met the criteria proceeded to the full-text assessment. The selected studies were carefully revised to ensure the exclusion of studies with repeated populations.

The data extraction was performed independently by two authors (J.C.M. and J.R.N.). A purposely built form was designed to collect relevant information including the year of publication, center of recruitment, country, study design, recruitment period, number of participants undergoing carotid endarterectomy, age, percentage of males, frequency of carotid symptomatic status and cardiovascular comorbidities, and data on the surgical setting, including the presence of contralateral disease, anticoagulation and antiplatelet therapies, the type of surgery, and the number of carotids operated on—Table 1, Table 2 and Table 3. Table 4 and Table 5 summarize the short-term and long-term clinical outcomes following CEA across the analyzed studies.

### 2.4. Assessment of Study Quality

Concerning the qualitative assessment, the National Heart, Lung, and Blood Institute (NHLBI) Study Quality Assessment Tool for observational cohort and cross-sectional studies (2021) was used to evaluate the methodological quality of the included studies [17]. This assessment was independently performed by two authors (J.C.M. and J.R.N.), and any disagreements were resolved through the involvement of a third author (A.P.N.). The included articles’ quality of evidence was assessed using the Grading of Recommendations, Assessment, Development, and Evaluation (GRADE) approach. The articles were classified into four levels of quality (high, moderate, low, and very low) [18].

## 3. Results

### 3.1. The Search Results

**Figure 1:**
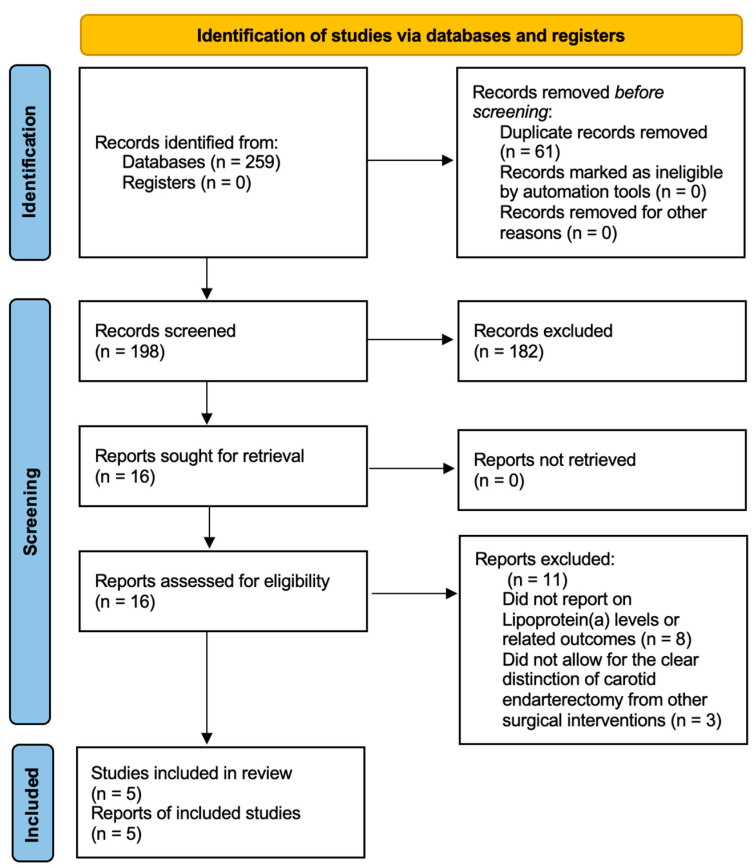
PRISMA 2020 flow diagram for systematic reviews.

After the database search and duplicate exclusion, 198 studies were screened. Following their selection based on their titles and abstracts, 182 studies were excluded. All of the remaining studies were accessible. A total of 16 studies were considered eligible for full-text review, after which point 11 studies were excluded, 8 because they did not report on Lp(a) levels or related outcomes and 3 because they did not allow for the clear distinction of CEA from other surgical interventions. Thus, five published articles were included in this systematic review (Figure 1).

### 3.2. Description of the Studies

This systematic review included five studies, all of which were observational cohort studies [4,8,9,19,20], with three of them being prospective (Table 1). In total, 1450 patients were evaluated, with each study including between 67 and 944 participants. The percentage of male gender ranged from 37.22% to 68.96%. The demographic data and comorbidities of the populations included in these studies were collected and are presented in Table 2 and Table 3.

In this systematic review, an association was identified between elevated Lp(a) levels and a significant increase in the risk of MACEs within 30 days following CEA. Specifically, Waissi et al. (2020) [4], in a Cox regression analysis of a cohort of 944 patients (with a 3-year follow-up time), revealed that elevated Lp(a) levels (>137 nmol/L; >80th percentile of the cohort) were associated with a hazard ratio of 2.05 (95% CI: 1.01–4.17) (adjusted for age and sex) compared with patients with low Lp(a) levels for MACEs within 30 days. This association was primarily driven by periprocedural stroke because 28 out of the 35 MACEs recorded in the first 30 days postop were strokes. This study also found no significant association between elevated Lp(a) levels and MACEs occurring between 30 days and 3 years post-surgery [4] (Table 4 and Table 5).

Additionally, Rigamonti et al. (2018) [8], in a cohort of 180 patients, showed that serum Lp(a) levels ≥ 10 mg/dL predict ACSs within 24 months following CEA, with an adjusted hazard ratio of 8.504 (95% CI: 1.932–37.425; *p* = 0.005) (adjusted for cardiovascular risk factors (CVRFs)). Here, the Lp(a) threshold value was based on previous studies that associated levels < 10 mg/dL with a low cardiovascular disease (CVD) risk. This study did not find a significant association between Lp(a) levels and histological inflammatory parameters of carotid plaques such as lipids, collagen, smooth muscle cells (SMCs), macrophages, neutrophils, and matrix metalloproteinase 9 (MMP-9). They suggested that Lp(a) levels had no association with histological signs of inflammation in carotid plaques [8] (Table 5).

Woźniak et al. (2024) [19] assessed intraplaque characteristics associated with plaque instability after CEA, including oxidized LDL cholesterol (ox-LDL), MMP-9, and 8-hydroxy-2′-deoxyguanosine (8-OHdG), and demonstrated that these parameters were significantly elevated in patients with unstable atherosclerotic plaques compared to those without, and crucially, this association was independent of Lp(a) levels (≥125 nmol/L or <125 nmol/L), highlighting the lack of influence of Lp(a) on plaque instability [19].

Carotid restenosis was assessed by Salenius et al. (1989) [20] in a cohort of 116 patients 28 to 209 months after CEA, and their results suggested low Lp(a) levels (<80 mg/L) were associated with a high frequency of restenosis, and the groups with intermediate (80–269 mg/L) and elevated levels (>269 mg/L) had low and average rates, respectively [20]. Additionally, Stinson et al. (1995) [9] found no association between high Lp(a) levels and the risk of restenosis after CEA over a follow-up period of 24 to 168 months in a cohort of 143 patients [9].

The measurement thresholds for elevated Lp(a) levels also differed, with the cut-off points ranging from 10 mg/mL to 270 mg/L and 125 nmol/L to 137 nmol/L. The measurement techniques spanned from latex-enhanced particle immunoturbidimetric assays to enzyme-linked immunosorbent assays or nephelometric assays, showcasing the variability in both the assays’ sensitivity and the reference ranges. Two studies did not specify the measurement methods (Table 6). 

The covariates included in the adjusted models across the analyzed studies are described in Supplemental Table S3. Common variables considered were demographic factors like age and sex, clinical parameters such as CVRFs, dyslipidemia, and coronary artery disease, and markers like oxidized LDL and MMP-9. Some studies incorporated comorbid conditions such as diabetes, hypertension, obesity, and smoking. However, two studies did not perform a multivariable analysis [9,20].

In this systematic review, the data available on Lp(a) and carotid artery disease were limited, with significant heterogeneity in the study designs and a lack of standardization in reporting the outcomes, making it challenging to meta-analyze the results.

### 3.3. Study Quality

**Figure 2:**
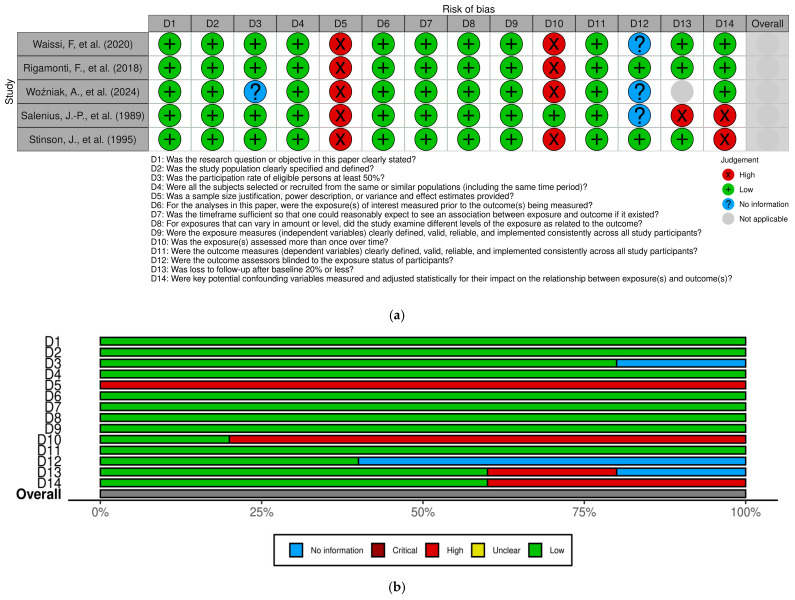
(**a**) The risk of bias in the selected studies [4,8,9,19,20]. (**b**) Overall judgment per evaluated item.

The risk of bias in the selected articles is presented in Figure 2a, while the overall assessment for each item related to observational cohorts is shown in Figure 2b.

All observational cohorts exhibited an overall low risk of bias. The factors most commonly linked to a high risk of bias in these cohorts were the sample size justification, the power analysis, and the assessment of exposure over time.

## 4. Discussion

These studies demonstrated that elevated levels of Lp(a) are associated with an increased risk of various cardiovascular diseases, including CAD, PAD, and ischemic stroke [6,21]. As was seen above, elevated Lp(a) levels interfere with fibrinolysis by mimicking plasminogen. This leads to the formation of more resistant clots, which contributes to a prothrombotic state that increases the risk of thromboembolic events, such as ischemic stroke and acute coronary syndromes [5,22].

This systematic review aggregated the evidence concerning Lp(a) levels and the outcomes after CEA. Based on the limited number of included studies, there was evidence to support an association of high Lp(a) levels with MACEs at 30 days of follow-up (mostly stroke) [4] and ACSs within 2 years after CEA [8]. On the other hand, Lp(a) levels were not associated with plaque instability and/or inflammation [19] or carotid restenosis [9,20]. Periprocedural stroke is a significant complication of this surgery that substantially reduces the beneficial effects of the procedure [23,24] and highlights the potential role of Lp(a) in thrombosis.

Lp(a) levels are genetically determined, and to date, no dietary modifications or medical therapies have been proven to be effective in lowering their concentration—while niacin and estrogens have been shown to significantly reduce Lp(a) levels, however, their use is not recommended due to their unfavorable safety profile [9,25]. These factors partly explain the limited research.

The Lp(a) measurement methods and the thresholds used to define elevated levels varied across all included studies. This heterogeneity underscores the challenge of standardizing Lp(a) values, as different assays and cut-off values can impact the comparability of the findings. Additionally, the lack of specification of the measurement methods in two of these studies highlights a gap in methodological transparency which may further affect the comparability of the results. The absence of a universally accepted threshold for elevated Lp(a) complicates the interpretation of its clinical significance and its role as a prognostic marker for CEA outcomes. Establishing a consistent outcome-based cut-off value through future research and consensus guidelines is key to enhancing the clinical applicability of Lp(a) measurements.

The assessment of the impact of Lp(a) in specific subgroups of patients undergoing CEA is limited. Although the primary analysis of the data does not reveal an overall association between elevated Lp(a) levels and adverse postoperative outcomes, some studies have attempted to address this question through different approaches. In the study by Waissi et al. (2020) [4], the results were adjusted for age and sex, which can be interpreted as a form of control for potential confounding factors but not as a formal subgroup analysis. Rigamonti et al. (2018) [8] compared the outcomes between patients with and without elevated Lp(a) levels and adjusted the results for CVRFs. The remaining studies included in this review did not perform analyses that allowed for the assessment of the impact of Lp(a) on specific subgroups of patients with diabetes, hypertension, or other relevant conditions. The absence of robust data on specific subgroups limits our ability to identify populations that may benefit from more specific therapeutic interventions. Future studies should be focused on evaluating the impact of Lp(a) in subgroups stratified by sex, age, symptomatic status, and the presence of relevant comorbidities.

Despite the absence of a direct association between Lp(a) and adverse outcomes in all patients undergoing CEA, the results of this review raise questions about the role of Lp(a) in clinical practice. The identification of patients with elevated Lp(a) levels may warrant a deeper assessment of cardiovascular risk, including the evaluation of other risk factors and biomarkers. In patients with elevated Lp(a) and other cardiovascular risk factors, it may be appropriate to intensify secondary prevention measures. Furthermore, the development of specific therapies to reduce Lp(a) levels may represent a promising strategy for improving the outcomes in patients undergoing CEA.

An important limitation of this review is the low number of and low data from the included primary studies, which prevented us from conducting a meta-analysis. Most of the studies included in this systematic review had small sample sizes, often lacking sample size justifications and power descriptions, contributing to low precision in the results. Additionally, significant heterogeneity was observed among the reported outcomes. There is a notable lack of research specifically addressing Lp(a) levels and their impact after carotid surgery. Thus, considering the diverse effects of Lp(a) on the human body discussed in this review, further research on this topic is essential, including large-size prospective cohort studies.

## 5. Conclusions

This systematic review suggests an association of Lp(a) levels with MACEs and ACSs after CEA although no association with restenosis and carotid plaque inflammation and/or instability. Despite its limitations, this review provides valuable insights into the prognostic value of elevated Lp(a) levels as a potential predictor of MACEs in patients undergoing CEA. It calls attention to the importance of further research to enhance patient outcomes, optimize care, and define its role in patients undergoing carotid surgery.

## Figures and Tables

**Table 1:** Summary of the studies included in the systematic review, detailing the author, year of publication, study design, recruitment location, recruitment period, number of participants, and quality assessment using the GRADE system.

Author	Journal	Publication Year	Study Design	Study Center	Recruitment Period	Sample Size (Patients)	GRADE Evaluation
Waissi F. et al.	Stroke (AHA Journal)	2020	Prospective cohort	St. Antonius Hospital Nieuwegein, University Medical Center Utrecht and Amsterdam University Medical Centers, the Netherlands	2002–2016	944	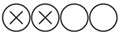 Low
Rigamonti F. et al.	European Journal of Clinical Investigation	2018	Prospective cohort (sub-study)	Hospital San Martino, Italy	2008–2012	180	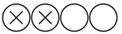 Low
Woźniak A. et al.	Journal of Cellular and Molecular Medicine	2024	Prospective cohort	Hospital M. Copernicus, Poland	2018–2019	67	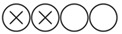 Low
Salenius J-P. et al.	European Journal of Vascular Surgery	1989	Retrospective cohort	University Central Hospital of Tampere, Finland	1970–1984	116	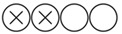 Low
Stinson J. et al.	Journal of Vascular Surgery	2009	Retrospective, observational study	Trinity Centre for Health Sciences, St. James’s Hospital, Ireland	1981–1995	143	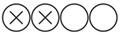 Low

**Table 2:** Demographic data and comorbidities of the population samples in each study (NA: unavailable data; Lp(a): lipoprotein (a); SD: standard deviation).

Author	Mean Age	Age SD	Male n (%)	Hypertension n (%)	Dyslipidemia n (%)	Obesity n (%)	Diabetes Mellitus n (%)	Chronic Heart Failure (CHF) n (%)	Smoking History n (%)	Coronary Artery Disease n (%)	Peripheral Artery Disease n (%)	Under Antiplatelet Therapy n (%)	Symptomatic n (%)
Waissi F. et al.	69.7	9	651 (68.96)	668 (70.76)	753 (79.77)	NA	209 (22.14)	NA	321 (34.00)	268 (28.39)	182 (19.28)	833 (88.24)	825 (87.39)
Rigamonti F. et al.	72 (not elevated Lp(a)); 74 (elevated Lp(a))	NA	67 (37.22)	126 (70.00)	101 (56.11)	NA	38 (21.11)	NA	40 (22.22)	34 (18.89)	NA	139 (77.22)	44 (24.44)
Woźniak A. et al.	71	NA	35 (52.24)	52 (77.61)	NA	5 (7.46)	15 (22.39)	NA	29 (43.28)	NA	NA	NA	NA
Salenius J-P. et al.	57	NA	79 (68.10)	68 (58.62)	74 (63.79)	33 (28.45)	13 (11.21)	NA	88 (75.86)	57 (49.14)	47 (40.52)	NA	100 (86.21)
Stinson J. et al.	69.5	NA	92 (64.33)	NA	NA	NA	NA	NA	NA	NA	NA	NA	NA

**Table 3:** Essential data regarding the surgical setting: presence of contralateral disease, anticoagulant and antiplatelet therapies, type of surgery, and number of carotids operated on (NA: unavailable data).

Author	Exclusion Criteria	Intervention	Number of Carotids Operated on per Person	Antiplatelet Therapy n (%)	Dual Antiplatelet Therapy n (%)	Anticoagulation n (%)	Contralateral Stenosis n (%)
Waissi F. et al.	Carotid endarterectomy for restenosis	All carotid endarterectomy	1	NA	NA	NA	823 (87.18)
Rigamonti F. et al.	The absence of angiographic visualization of the symptomatic artery, intracranial stenosis more clinically significant than the cervical lesion, other diseases that limited life expectancy to less than five years, cerebral infarction that resulted in the loss of useful function in the affected arterial territory, non-atherosclerotic carotid disease, cardiac lesions likely to cause cardioembolism, or a history of ipsilateral endarterectomy.	All carotid endarterectomy	1	NA	NA	NA	NA
Woźniak A. et al.	Causes of non-vascular ischemic stroke, prior hemorrhagic or lacunar strokes, brain disorders, atrial fibrillation treatment, active infection or inflammation, autoimmune conditions, hematological and oncological disorders, severe renal or hepatic failure, venous thrombosis, myocardial infarction or surgical procedures within one year before inclusion, the use of anti-inflammatory or immunosuppressive drugs within 6 months before examination, malnutrition, poisoning, or alcohol and psychoactive substance abuse history.	All carotid endarterectomy	1	NA	NA	NA	NA
Salenius J-P. et al.	NA	All carotid endarterectomy	90 persons—1 carotid 22 persons—bilateral 4 persons—two separate endarterectomies on the same side	32 (27.6)	ASA–dipyridamole combination: 27 (23.3)	28 (24.1)	NA
Stinson J. et al.	NA	All carotid endarterectomy	1	NA	NA	NA	NA

**Table 4:** Short-term (up to 30-day) adverse outcomes following carotid endarterectomy in the included studies. Reported events include stroke, acute myocardial infarction (AMI), cardiovascular-related death, and MACEs, with the definitions varying among studies (NA: unavailable data. HR: hazard ratio. AMI: acute myocardial infarction. MACEs: major adverse cardiovascular events).

Author	Stroke—30 Days n (%)	AMI—30 Days n (%)	Cardiovascular-Related Deaths in 30 Days n (%)	30-Day MACEs n (%)	30-Day MACEs (Elevated Lp(a) Levels) HR	MACE Definitions
Waissi F. et al.	28 (2.75)	6 (0.64)	1 (0.10)	35 (3.71)	HR of 2.12 (95% CI: 1.05–4.27) HR of 2.05 (95% CI: 1.01–4.17) (adjusted for age and sex)	Myocardial infarction, stroke, or cardiovascular death
Rigamonti F. et al.	NA	NA	NA	NA	NA	NA
Woźniak A. et al.	NA	NA	NA	NA	NA	NA
Salenius J-P. et al.	NA	NA	NA	NA	NA	NA
Stinson J. et al.	NA	NA	NA	NA	NA	NA

**Table 5:** Long-term outcomes after carotid endarterectomy in the included studies, with follow-up periods ranging from 24 to 209 months. Data include stroke, acute myocardial infarction, cardiovascular-related death, and the incidence of MACEs over time (NA: unavailable data. AMI: acute myocardial infarction. MACE: Major adverse cardiovascular events. HR: Hazard Ratio. Lp(a): lipoprotein (a). ACS: acute coronary syndrome.).

Author	Follow-Up Time	AMI >30 Days n (%)	AMI Long-Term Follow-Up	Stroke > 30 Days n (%)	Stroke Long-Term Follow-Up	Cardiovascular-Related Death >30 Days n (%)	MACEs in Long-Term Follow-Up	MACEs > 30 Days n (%)	Other Outcomes	Long-Term All-Cause Mortality n (%)
Waissi F. et al.	3 years	21 (2.22)	27 (2.86)	35 (3.71)	63 (6.67)	11 (1.17)	102 (10.8%) High Lp(a) levels: HR: 1.54 (95% CI: 1.00–2.39). HR: 1.69 (95% CI: 1.07–2.66) (adjusted for risk factors).	“No significant association between high Lp(a) levels and MACE from time point 30 days to 3 years onward”	NA	NA
Rigamonti F. et al.	24 months	NA	ACS long-term follow-up: High Lp(a)— HR: 6.490 (95% CI: 1.550–27.160, *p* = 0.010) HR: 8.504 (95% CI: 1.932–37.425], *p* = 0.005) (adjusted for CV risk factors)	NA	NA	NA	NA	NA	NA	NA
Woźniak A. et al.	NA	NA	NA	NA	NA	NA	NA	NA	Regardless of serum Lp(a) levels, in patients with unstable atherosclerotic plaques, other serum components such as ox-LDL, MMP-9, and 8-OHdG are higher than these values in patients without unstable plaques.	NA
Salenius J-P. et al.	28 to 209 months	NA	NA	NA	NA	NA	NA	NA	Low Lp(a) levels indicated a higher frequency of high-grade restenosis: low levels (<80 mg/L): 20.9%; intermediate levels (80–269 mg/L): 7.5%; high levels (>270 mg/L): 12.0%; *p* = 0.10.	NA
Stinson J. et al.	24 to 168 months	NA	NA	NA	NA	NA	NA	NA	No association was found between Lp(a) concentrations and risk of restenosis.	NA

**Table 6:** Lp(a) values and measurements.

Author	Lp(a) Elevated	Measurement
Waissi F. et al.	>137 nmol/L	Lp(a) concentrations were measured using a latex-enhanced particle immunoturbidimetric assay with the Cobas c702 analyzer and the LPA2 Tinaquant Lp(a) Gen.2 kit. The method involved the agglutination of Lp(a) in the serum samples using latex particles coated with anti-Lp(a) antibodies. The resulting precipitate was quantified turbidimetrically at 800/660 nm. The assay has a measuring range of 7 to 240 nmol/L.
Rigamonti F. et al.	> or = 10 mg/mL	Serum Lp(a) levels were measured using a nephelometric assay.
Woźniak A. et al.	> or = 125 nmol/L	Immunoturbidimetric assay.
Salenius J-P. et al.	> or = 270 mg/L	NA
Stinson J. et al.	Mean Lp(a) levels in patients with carotid stenosis: 390 ± 40.2 mg/L Mean Lp(a) levels in controls: 142 ± 29.7 mg/L	Lipoprotein (a) concentrations were determined using an enzyme-linked immunosorbent assay (ELISA).

Notes. NA: unavailable data; Lp(a): lipoprotein (a).

## Data Availability

The original contributions presented in this study are included in the article/Supplementary Material. Further inquiries can be directed to the corresponding author(s).

## Supplementary Materials

The following supporting information can be downloaded at https://www.mdpi.com/article/10.3390/jcm14072253/s1. Supplemental Table S1: PRISMA checklist details; Supplemental Table S2: Search query—keywords; Supplemental Table S3: Covariates used in the adjusted models.

## Abbreviations

The following abbreviations are used in this manuscript:

ACS	Acute coronary syndrome
AMSTAR	A Measurement Tool to Assess Systematic Reviews
Apo(a)	Apolipoprotein(a)
Apo B100	Apolipoprotein B100
BMT	Best medical therapy
CAD	Coronary artery disease
CEA	Carotid endarterectomy
CI	Confidence interval
CVD	Cardiovascular disease
CVRF	Cardiovascular risk factor
GRADE	Grading of Recommendations, Assessment, Development, and Evaluation
LDL	Low-density lipoprotein
Lp(a)	Lipoprotein(a)
MACE	Major adverse cardiovascular event
MMP-9	Matrix metalloproteinase-9
NHLBI	National Heart, Lung, and Blood Institute
ox-LDL	Oxidized low-density lipoprotein
PAD	Peripheral arterial disease
PRISMA	Preferred Reporting Items for Systematic Reviews and Meta-Analyses
SMCs	Smooth muscle cells
