# Supplementary material for: The Impact of Elevated Lipoprotein (a) Levels on Postoperative Outcomes in Carotid Endarterectomy: A Systematic Review
Source: J Clin Med. 2025 Mar 26;14(7):2253. doi: 10.3390/jcm14072253 (PMC11989823; doi:10.3390/jcm14072253)
Supplement: Supplementary file 1 [file jcm-14-02253-s001.zip › jcm-3502888-supplementary.pdf]

Supplemental Table 1: PRISMA checklist details.

Section and Topic	Item #	Checklist item	Location where item is reported
**TITLE**			
Title	1	Identify the report as a systematic review.	Page 1: "The Impact of Elevated Lipoprotein(a) Levels on Postoperative Outcomes in Carotid Endarterectomy: A Systematic Review"
**ABSTRACT**			
Abstract	2	See the PRISMA 2020 for Abstracts checklist.	Page 3, first paragraph of the section "2. Materials and Methods"
**INTRODUCTION**			
Rationale	3	Describe the rationale for the review in the context of existing knowledge.	Pages 2, last 2 paragraphs of the "Introduction": "Furthermore, previous studies have identified Lp(a) as an independent risk factor for the development of coronary artery disease, peripheral vascular disease, and the recurrence of cardiovascular events such as acute myocardial infarction and stroke.<br>Thus, this systematic review aims to evaluate the impact of elevated Lp(a) levels on postoperative outcomes in patients undergoing CEA."
Objectives	4	Provide an explicit statement of the objective(s) or question(s) the review addresses.	Page 2 "Thus, this systematic review aims to evaluate the impact of elevated Lp(a) levels on postoperative outcomes in patients undergoing CEA."
**METHODS**			
Eligibility criteria	5	Specify the inclusion and exclusion criteria for the review and how studies were grouped for the syntheses.	Page 3 in the selection criteria section.
Information sources	6	Specify all databases, registers, websites, organisations, reference lists and other sources searched or consulted to identify studies. Specify the date when each source was last searched or consulted.	Page 3 "A systematic search was performed in four databases – PubMed, Scopus, Web of Science and Cochrane

Section and Topic	Item #	Checklist item	Location where item is reported
			Library, in October 2024."
Search strategy	7	Present the full search strategies for all databases, registers and websites, including any filters and limits used.	Supplemental table 1.
Selection process	8	Specify the methods used to decide whether a study met the inclusion criteria of the review, including how many reviewers screened each record and each report retrieved, whether they worked independently, and if applicable, details of automation tools used in the process.	Page 3 "After removing duplicates, two authors (JCM and JRN) independently selected studies, with any disagreements resolved by a third author (APN). The initial screening was based on titles and abstracts, and studies that met the criteria proceeded to full text assessment. The selected studies were carefully revised to ensure the exclusion of studies with repeated populations."
Data collection process	9	Specify the methods used to collect data from reports, including how many reviewers collected data from each report, whether they worked independently, any processes for obtaining or confirming data from study investigators, and if applicable, details of automation tools used in the process.	Page 3 "Data extraction was performed independently by two authors (JCM and JRN). A purposely built form was designed to collect the relevant information".
Data items	10a	List and define all outcomes for which data were sought. Specify whether all results that were compatible with each outcome domain in each study were sought (e.g. for all measures, time points, analyses), and if not, the methods used to decide which results to collect.	Tables 4 and 5.
	10b	List and define all other variables for which data were sought (e.g. participant and intervention characteristics, funding sources). Describe any assumptions made about any missing or unclear information.	Page 3 "A purposely built form was designed to collect the relevant information including the year of publication, country, and center of recruitment, study design, recruitment time, number of participants undergoing carotid endarterectomy, participants' age, percentage of males, frequency of cardiovascular comorbidities and carotid symptomatic status, data related to the surgical setting, including the presence of contralateral disease, antiplatelet and anticoagulation therapies, type of surgery, and the number of carotids operated - Table 1, Table 2 and Table 3."

Section and Topic	Item #	Checklist item	Location where item is reported
Study risk of bias assessment	11	Specify the methods used to assess risk of bias in the included studies, including details of the tool(s) used, how many reviewers assessed each study and whether they worked independently, and if applicable, details of automation tools used in the process.	Page 10 "Concerning qualitative assessment, the National Heart, Lung, and Blood Institute (NHLBI) Study Quality Assessment Tool for observational cohort and cross-sectional studies"
Effect measures	12	Specify for each outcome the effect measure(s) (e.g. risk ratio, mean difference) used in the synthesis or presentation of results.	Tables 4 and 5.
Synthesis methods	13a	Describe the processes used to decide which studies were eligible for each synthesis (e.g. tabulating the study intervention characteristics and comparing against the planned groups for each synthesis (item #5)).	Not applicable.
	13b	Describe any methods required to prepare the data for presentation or synthesis, such as handling of missing summary statistics, or data conversions.	Not applicable.
	13c	Describe any methods used to tabulate or visually display results of individual studies and syntheses.	Not applicable.
	13d	Describe any methods used to synthesize results and provide a rationale for the choice(s). If meta-analysis was performed, describe the model(s), method(s) to identify the presence and extent of statistical heterogeneity, and software package(s) used.	Not applicable.
	13e	Describe any methods used to explore possible causes of heterogeneity among study results (e.g. subgroup analysis, meta-regression).	Not applicable.
	13f	Describe any sensitivity analyses conducted to assess robustness of the synthesized results.	Not applicable.
Reporting bias assessment	14	Describe any methods used to assess risk of bias due to missing results in a synthesis (arising from reporting biases).	Not applicable.
Certainty assessment	15	Describe any methods used to assess certainty (or confidence) in the body of evidence for an outcome.	Not applicable.
**RESULTS**			
Study selection	16a	Describe the results of the search and selection process, from the number of records identified in the search to the number of studies included in the review, ideally using a flow diagram.	Figure 1.
	16b	Cite studies that might appear to meet the inclusion criteria, but which were excluded, and explain why they were excluded.	Not applicable.
Study characteristics	17	Cite each included study and present its characteristics.	Section "3.2. Description of studies", pages 11-12 and tables 1-5.
Risk of bias in	18	Present assessments of risk of bias for each included study.	Figures 2a and 2b.

Section and Topic	Item #	Checklist item	Location where item is reported
studies			
Results of individual studies	19	For all outcomes, present, for each study: (a) summary statistics for each group (where appropriate) and (b) an effect estimate and its precision (e.g. confidence/credible interval), ideally using structured tables or plots.	Not applicable.
Results of syntheses	20a	For each synthesis, briefly summarise the characteristics and risk of bias among contributing studies.	Not applicable.
	20b	Present results of all statistical syntheses conducted. If meta-analysis was done, present for each the summary estimate and its precision (e.g. confidence/credible interval) and measures of statistical heterogeneity. If comparing groups, describe the direction of the effect.	Not applicable.
	20c	Present results of all investigations of possible causes of heterogeneity among study results.	Not applicable.
	20d	Present results of all sensitivity analyses conducted to assess the robustness of the synthesized results.	Not applicable.
Reporting biases	21	Present assessments of risk of bias due to missing results (arising from reporting biases) for each synthesis assessed.	Not applicable.
Certainty of evidence	22	Present assessments of certainty (or confidence) in the body of evidence for each outcome assessed.	Not applicable.
**DISCUSSION**			
Discussion	23a	Provide a general interpretation of the results in the context of other evidence.	Summarized in the “Discussion” section (pages 12-13) and in tables 4 and 5.
	23b	Discuss any limitations of the evidence included in the review.	Pages 3, last three paragraphs of the “Discussion” section.
	23c	Discuss any limitations of the review processes used.	Not applicable.
	23d	Discuss implications of the results for practice, policy, and future research.	Page 13, “Conclusion” section.
**OTHER INFORMATION**			
Registration and protocol	24a	Provide registration information for the review, including register name and registration number, or state that the review was not registered.	Page 3, section “2. Material and Methods”, “The review protocol has been registered at Prospero (reference: CRD42024603037).”
	24b	Indicate where the review protocol can be accessed, or state that a protocol was not prepared.	The review protocol can be accessed on https://www.crd.york.ac.uk/prospero/display_record.php?ID=CRD42024603037.

Section and Topic	Item #	Checklist item	Location where item is reported
			Reference number is CRD42024603037.
	24c	Describe and explain any amendments to information provided at registration or in the protocol.	Not applicable.
Support	25	Describe sources of financial or non-financial support for the review, and the role of the funders or sponsors in the review.	Page 14, section "Funding", "This article was supported by National Funds through FCT - Fundação para a Ciência e a Tecnologia, I.P., within CINTESIS, R&D Unit (reference UIDB/4255/2020)."
Competing interests	26	Declare any competing interests of review authors.	Page 14, section "Conflict of Interest Statement", "Mariana Fragão Marques is an employee of Novo Nordisk."
Availability of data, code and other materials	27	Report which of the following are publicly available and where they can be found: template data collection forms; data extracted from included studies; data used for all analyses; analytic code; any other materials used in the review.	Not applicable.

From: Page MJ, McKenzie JE, Bossuyt PM, Boutron I, Hoffmann TC, Mulrow CD, et al. The PRISMA 2020 statement: an updated guideline for reporting systematic reviews. BMJ 2021;372:n71. doi: 10.1136/bmj.n71

Supplemental table 2. Search query – keywords

Database	Fields	Search Terms
PubMed	MeSH Terms	none
	Free text words	high-density lipoprotein(a) (All fields)<br>lipoprotein(a) (All fields)<br>Lp(a) (All fields)<br>apo(a) (All fields)<br>apolipoprotein(a) (All fields)<br>LPA locus (All fields)<br>Endarterectomy (All fields)<br>Endarterectomy, Carotid (All fields)<br>carotid endarterectomy (All fields)<br>Carotid Endarterectomies (All fields)
	Limits	None
Scopus	Free text words	high-density lipoprotein(a) (Article title, Abstract, Keywords)<br>lipoprotein(a) (Article title, Abstract, Keywords)<br>Lp(a) (Article title, Abstract, Keywords)<br>apo(a) (Article title, Abstract, Keywords)<br>apolipoprotein(a) (Article title, Abstract, Keywords)<br>LPA locus (Article title, Abstract, Keywords)<br>Endarterectomy (Article title, Abstract, Keywords)<br>Endarterectomy, Carotid (Article title, Abstract, Keywords)<br>carotid endarterectomy (Article title, Abstract, Keywords)<br>Carotid Endarterectomies (Article title, Abstract, Keywords)
	Limits	None
Web of Science	Free text words	high-density lipoprotein(a) (All fields)<br>lipoprotein(a) (All fields)<br>Lp(a) (All fields)<br>apo(a) (All fields)<br>apolipoprotein(a) (All fields)<br>LPA locus (All fields)<br>Endarterectomy (All fields)<br>Endarterectomy, Carotid (All fields)<br>carotid endarterectomy (All fields)<br>Carotid Endarterectomies (All fields)
	Limits	None

Cochrane	Free text words	high-density lipoprotein(a) (Article title, Abstract, Keywords)<br>lipoprotein(a) (Article title, Abstract, Keywords)<br>Lp(a) (Article title, Abstract, Keywords)<br>apo(a) (Article title, Abstract, Keywords)<br>apolipoprotein(a) (Article title, Abstract, Keywords)<br>LPA locus (Article title, Abstract, Keywords)<br>Endarterectomy (Article title, Abstract, Keywords)<br>Endarterectomy, Carotid (Article title, Abstract, Keywords)<br>carotid endarterectomy (Article title, Abstract, Keywords)<br>Carotid Endarterectomies (Article title, Abstract, Keywords)
	Limits	None

Supplemental table 3. Covariates used in the adjusted models

**Author**	Covariables used in ajusted models
Waissi F. et al	Age and sex, CVRF
Rigamont F. et al	Gender, dyslipidemia, coronary artery disease
Woźniak A. et al	Ox-LDL, MMP-9; Diabetes mellitus, arterial hypertension, obesity, smoking, stroke
Salenius J-P. et al	NA
Stinson J. et al	NA

CVRF - cardiovascular risk factors; Ox-LDL – oxidized low-density lipoprotein; MMP-9 – matrix metalloproteinase-9; NA- unavailable data.
